# Granulomatous Lymphocytic Interstitial Lung Disease as the Initial Manifestation of Common Variable Immunodeficiency in a Young Adult

**DOI:** 10.7759/cureus.99208

**Published:** 2025-12-14

**Authors:** Pedro Sá Almeida, Ana Maria Carvalho, Rita G Magalhães, Tiago Silveira-Rosa, João Enes Silva

**Affiliations:** 1 Internal Medicine, Unidade Local de Saúde de Trás-os-Montes e Alto Douro, Chaves, PRT

**Keywords:** common variable immunodeficiency syndrome, hepatosplenomegaly, hypogammaglobulinaemia, immunoglobulin replacement therapy, interstitial lung diseases (ilds), non-necrotizing granuloma, young adult case

## Abstract

Common variable immunodeficiency (CVID) is a primary immunodeficiency that often goes unrecognized until adolescence or adulthood. Granulomatous-lymphocytic interstitial lung disease (GLILD) is a non-infectious pulmonary complication of CVID that can be subtle, sometimes presenting before significant infections or systemic symptoms. A 19-year-old female patient presented with mild flu-like symptoms. Examination revealed splenomegaly, and routine labs showed mild anaemia and thrombocytopenia. Despite rapid recovery from influenza A, persistent splenomegaly prompted further evaluation. Chest CT demonstrated multiple nodules, ground-glass opacities, and mediastinal lymphadenopathy, findings surprisingly disproportionate to her mild symptoms. Her history of recurrent childhood infections, combined with marked hypogammaglobulinemia and abnormal B-cell immunophenotyping (including increased CD21^low^ B cells), raised suspicion for CVID. Lung biopsy confirmed GLILD. She was started on immunoglobulin replacement therapy, and at follow-up, her pulmonary lesions remained stable with no new infections. This case highlights how CVID can present subtly, with imaging findings that far exceed the apparent clinical severity. GLILD may be the first clue to an underlying immunodeficiency, emphasizing the importance of a detailed history, immunologic testing, and histopathologic confirmation. Timely recognition allows for early initiation of therapy and close monitoring, potentially improving long-term outcomes. Even mild respiratory symptoms may mask serious underlying immune dysregulation. Clinicians should consider primary immunodeficiencies like CVID in young adults when imaging or lab findings are inconsistent with common infections, as early diagnosis can significantly impact management and prognosis.

## Introduction

Common variable immunodeficiency (CVID) is a heterogeneous primary immunodeficiency characterized by low immunoglobulin levels (IgG, IgA, and/or IgM), impaired antibody production, and a broad spectrum of clinical manifestations, including recurrent infections, autoimmunity, and lymphoproliferative disorders [1].

Among its non-infectious complications, granulomatous-lymphocytic interstitial lung disease (GLILD) is particularly significant, with 10-20% prevalence in CVID, and it is associated with a significantly worse prognosis [2]. GLILD manifests with lymphoid hyperplasia, non-necrotizing granulomas, and distinctive imaging patterns such as pulmonary nodules, ground-glass opacities, septal thickening, and hilar or mediastinal lymphadenopathy [3,4]. It often coexists with systemic lymphoproliferation, including hepatosplenomegaly and generalized lymphadenopathy, highlighting that pulmonary involvement is frequently part of broader immune dysregulation [2,3]. Diagnosing GLILD can be challenging, as imaging findings may overlap with infectious or malignant processes, and lung biopsy is not always feasible [5]. Treatment approaches vary widely, including corticosteroids, immunomodulators, and B-cell-targeted therapies, with no standardized guidelines [4].

We report the case of a 19-year-old woman whose imaging findings were suggestive of GLILD, together with hepatosplenomegaly and generalized lymphadenopathy, which led to an unexpected diagnosis of CVID. Notably, her pulmonary lesions were disproportionate to the mild severity of her respiratory symptoms, illustrating how subtle clinical presentations, even when childhood infections were previously overlooked, can mask underlying immune dysregulation. This case emphasizes the importance of careful evaluation and high clinical suspicion in young adults presenting with discordant imaging and clinical features.

## Case presentation

A 19-year-old female university student presented to the emergency department with flu-like symptoms. Physical examination revealed splenomegaly; peripheral lymphadenopathy was not palpable. Initial laboratory evaluation revealed mild microcytic anaemia and thrombocytopenia (Table 1).

**Table 1:** Initial laboratory evaluation with follow-up comparison CRP: C-reactive protein; MCH: mean corpuscular haemoglobin; MCV: mean corpuscular volume; MPV: mean platelet volume; TSAT: transferrin saturation; WBC: white blood cells; pCO2: partial pressure of carbon dioxide; pO2: partial pressure of oxygen

Parameters	Patient Values at Admission	Patient Values at Follow-up	Reference Ranges
Hemoglobin	11.20 g/dL	10.90 g/dL	12.00-16.00 g/dL
MCV	75.9 fL	76.3 fL	87.0-103.0 fL
MCH	24.3 pg	24.2 pg	27.0-33.0 pg
Ferritin	-	48.6 ng/mL	15.0-150.0 ng/mL
TSAT	-	7.1%	>20%
WBC count	4,660/µL	4,040/µL	4,000-11,000/µL
Neutrophils	3,400/µL	2,330/µL	1,500-8,000/µL
Lymphocytes	1,140/µL	1,230/µL	800-4,000/µL
Monocytes	90/µL	330/µL	0-1,200/µL
Eosinophils	20/µL	70/µL	0-300/µL
Basophils	0/µL	20/µL	0-300/µL
Platelet count	90,000/µL	109,000/µL	150,000-400,000/µL
MPV	10.8 fL	10.2 fL	7.4-10.9 fL
Reticulocyte count	-	0.086x10^12^/L	0.025-0.090x10^12^/L
pH (blood gas)	7.41	-	7.35-7.45
pCO2 (blood gas)	37 mmHg	-	35-45 mmHg
pO2 (blood gas)	84 mmHg	-	80-100 mmHg
sO2 (blood gas)	99%	-	95-100%
CRP	1.88 mg/dL	0.51 mg/dL	<0.5 mg/dL

**Figure 1:**
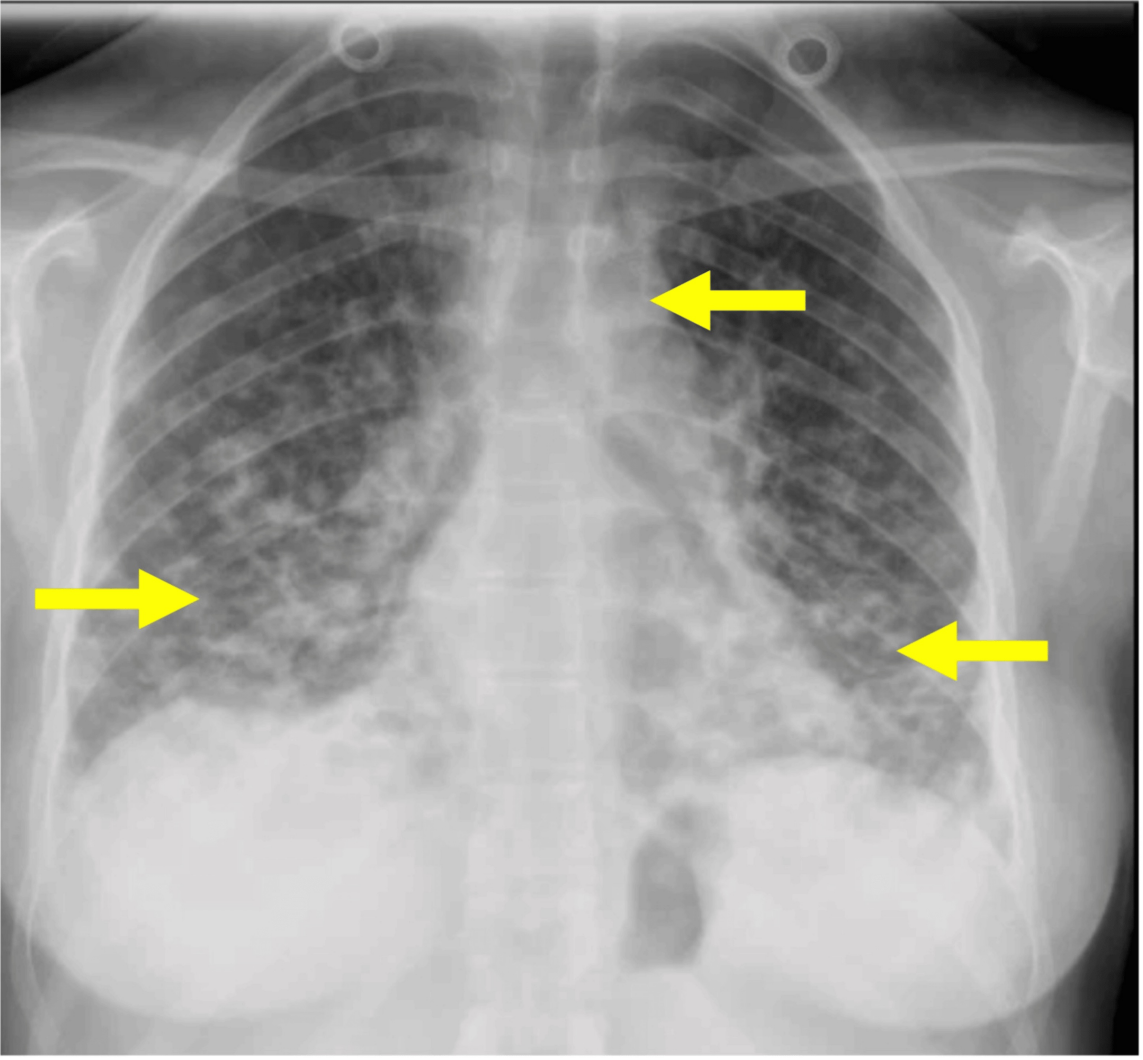
Posteroanterior chest X-ray Bibasal infiltrates and mediastinal enlargement (arrows)

Chest X-ray demonstrated bibasal infiltrates (Figure 1), although she exhibited no respiratory insufficiency with room air peripheral oxygen saturation (SpO2) of 99%. She was diagnosed with influenza A and treated with oseltamivir and supportive care. One week later, clinical and laboratory improvement was noted, but the previous findings of the chest X-ray and the persistence of splenomegaly prompted further evaluation.

**Figure 2:**
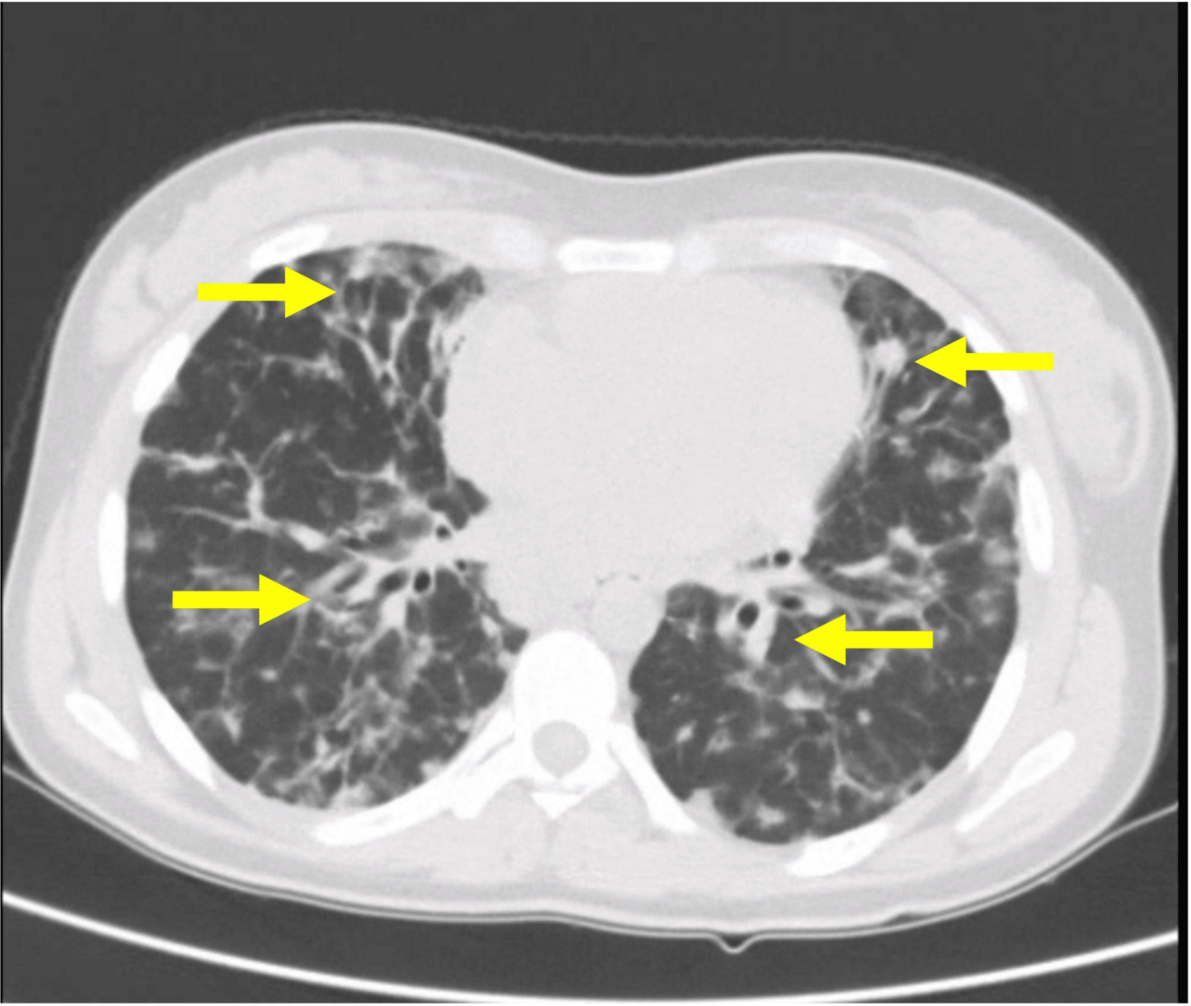
Axial plane of chest computed tomography Multiple centrilobular nodules, areas of ground-glass opacities, and subpleural consolidations, predominantly in basal lung segments (arrows)

**Figure 3:**
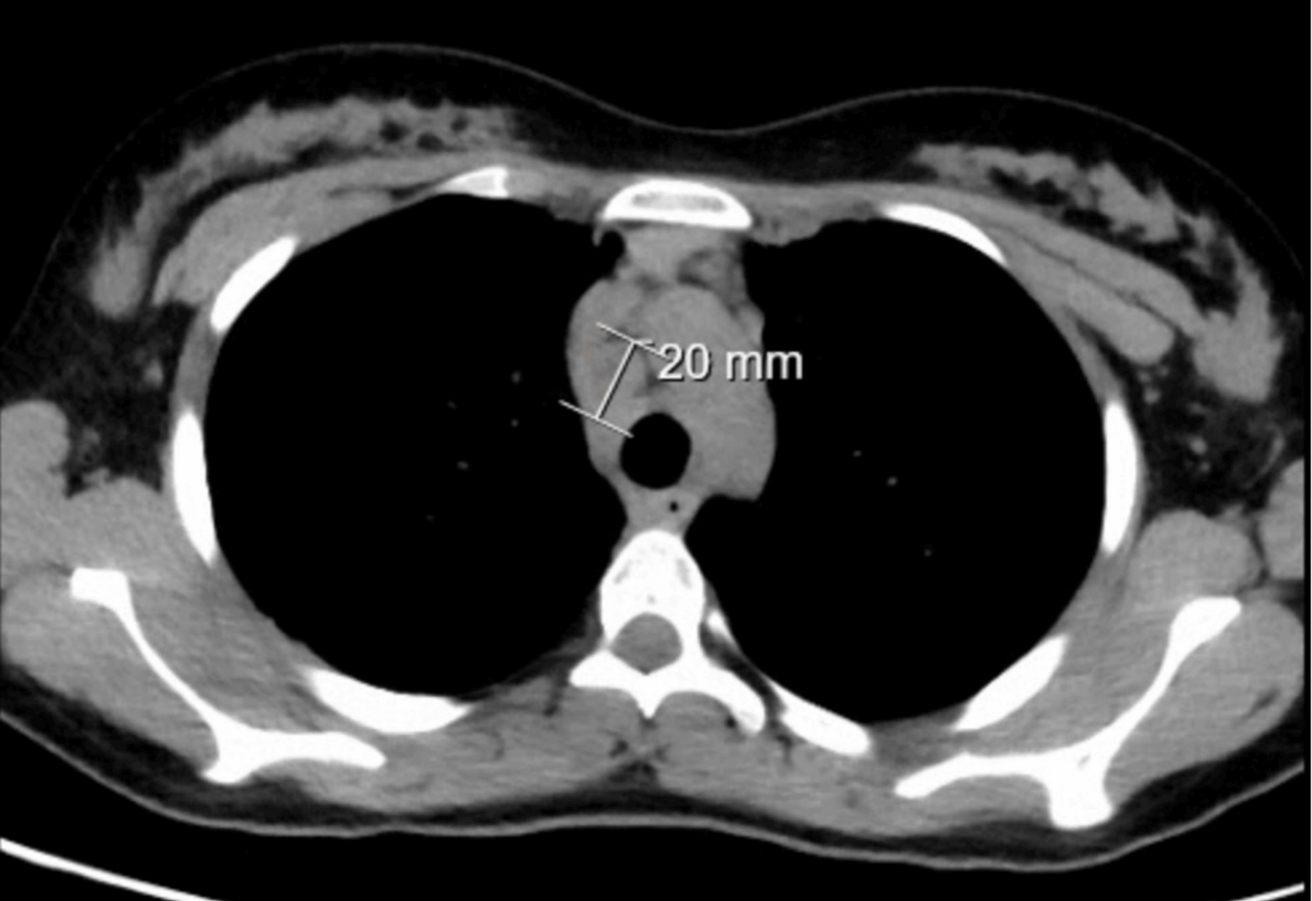
Axial plane of chest computed tomography in mediastinal view Mediastinal lymphadenopathy with nodes measuring up to 20 mm.

**Figure 4:**
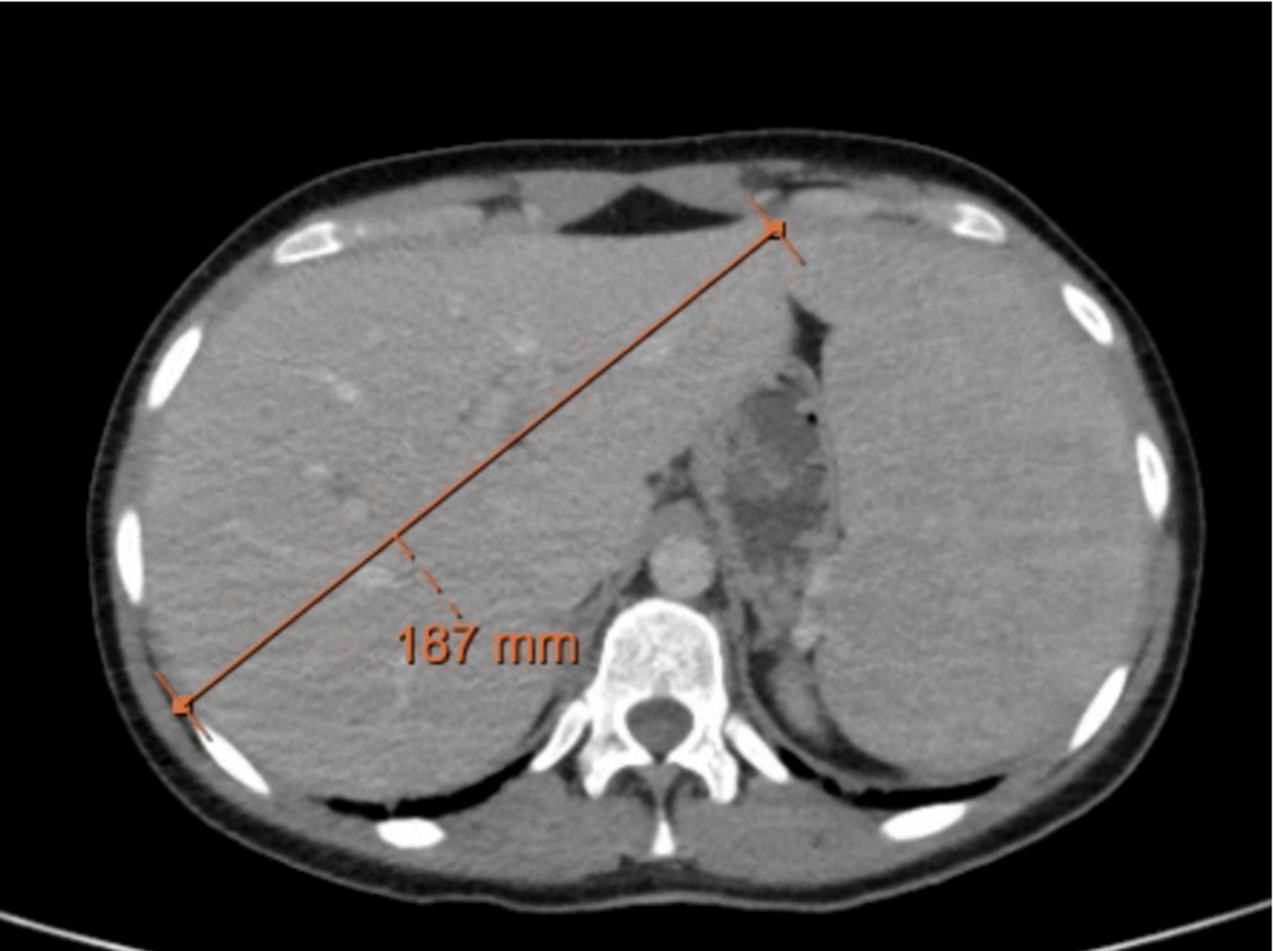
Axial plane of abdomen and pelvis computed tomography Heterogeneous hepatomegaly (18.7 cm)

**Figure 5:**
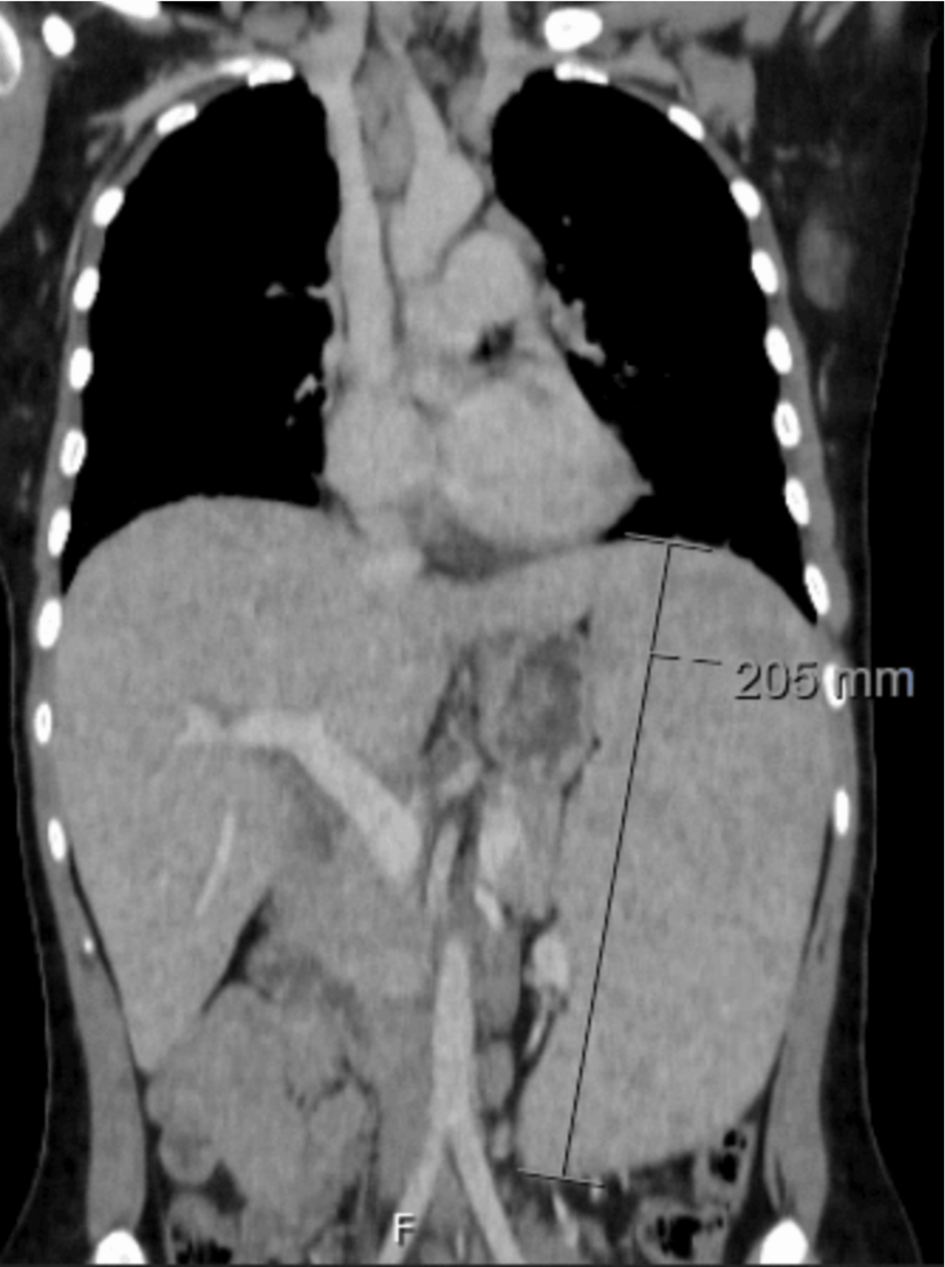
Axial abdomen and pelvis computerized tomography Heterogeneous splenomegaly (20.5 cm)

Chest computed tomography (CT) revealed multiple centrilobular nodules, areas of ground-glass opacities, and subpleural consolidations, predominantly in basal lung segments and the lingula (Figure 2). Mediastinal lymphadenopathy was present, with nodes measuring up to 20 mm (Figure 3). These findings, atypical for acute influenza, were considered disproportionate to the mild clinical symptoms, raising concern for underlying pathology. CT of the abdomen and pelvis revealed heterogeneous hepatosplenomegaly (Figures 4, 5). She denied systemic symptoms such as fever, night sweats, weight loss, diarrhoea, or pruritus.

**Figure 6:**
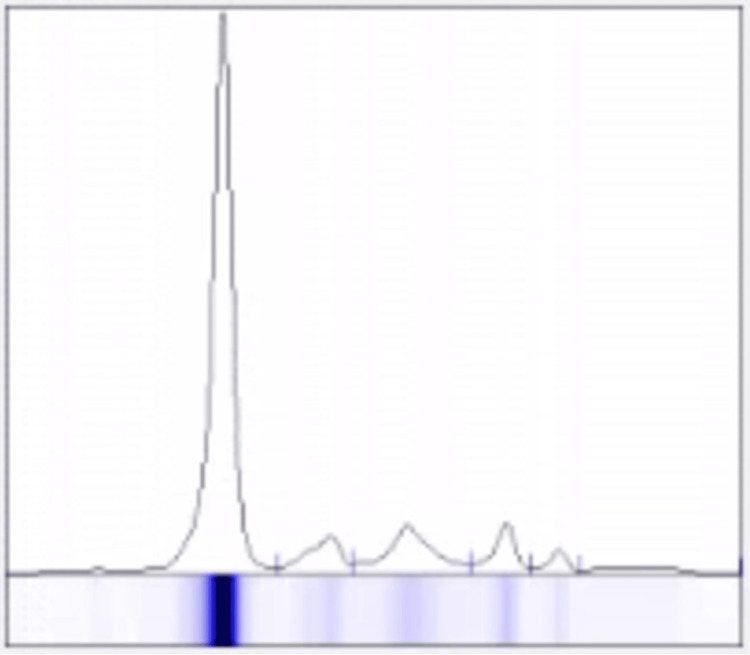
Serum protein electrophoresis graph

During outpatient follow-up, she reported mild, chronic fatigue, attributed to longstanding anaemia. Laboratory studies showed recurrent thrombocytopenia and iron deficiency anaemia (Table 1). Prior to admission, the patient underwent upper gastrointestinal endoscopy and colonoscopy, which were both normal, as prescribed by her general practitioner. Marked hypogammaglobulinemia was also noted (Table 2). Serum protein electrophoresis revealed no monoclonal spike (Figure 6). Additional investigations showed elevated angiotensin-converting enzyme (ACE) and β2-microglobulin, while inflammatory markers, autoantibodies, and infectious serologies, including HIV, hepatitis B and C, Epstein-Barr virus (EBV), cytomegalovirus (CMV), syphilis, toxoplasmosis, and atypical viruses, were negative (Table 2). Tuberculosis was also ruled out (Table 2).

**Table 2:** Further laboratory investigation ACE: angiotensin-converting enzyme; ANA: antinuclear antibodies; Anti-HBc: hepatitis B core antibody; Anti-HBs: hepatitis B surface antibody; Anti-HCV: hepatitis C antibody; cANCA: anti-neutrophil cytoplasmic antibodies; CMV: cytomegalovirus; EBV: Epstein-Barr virus; FTA-ABS: fluorescent treponemal antibody absorption; HBsAg: hepatitis B surface antigen; HIV: human immunodeficiency virus; Ig: immunoglobulin; SPE: serum protein electrophoresis; VCA: viral capsid antigen

Parameters	Patient Values	Reference Ranges
IgG	145 mg/dL	650-1500 mg/dL
IgA	<50 mg/dL	78-312 mg/dL
IgM	11 mg/dL	55-300 mg/dL
Albumin (SPE)	69.2%	54.6-66.6%
Alfa 1 globulin (SPE)	7.2%	3.2-6.0%
Alfa 2 globulin (SPE)	12.2%	7.0-11.6%
Beta 1 globulin (SPE)	6.3%	4.5-8.3%
Beta 2 globulin (SPE)	2.5%	2.9-5.7%
Gamma globulin (SPE)	2.6%	11.8-17.8%
Albumin/Globulin (SPE)	2.3	1.0-2.5
ACE	132 U/L	20-70 U/L
β2-microglobulin	5.86 mg/L	<2.53 mg/L
ANA	<1:160	<1:160
cANCA	<1:20	<1:20
HIV (3rd generation test)	Negative	
HBsAg	Negative	
Anti-HBc (Total)	Negative	
Anti-HBs	Negative	
Anti-HCV	Negative	
VCA (EBV) IgG	Negative	
VCA (EBV) IgM	Negative	
CMV IgG	Negative	
CMV IgM	Negative	
FTA-ABS (Syphilis)	Negative	
Toxoplasmosis IgG	Negative	
Toxoplasmosis IgM	Negative	
Adenovirus IgG	Negative	
Parvovirus B19 IgG/IgM	Negative	
Varicella-zoster IgG/IgM	Negative	
Rickettsia spp IgG/IgM	Negative	
DNA Tuberculosis	Negative	
Cultural Tuberculosis	Negative	

Her past medical history was notable for recurrent infections in childhood, including media otitis, respiratory infections, and gastrointestinal illnesses, as well as iron-deficiency anaemia related to menorrhagia, previously investigated with normal endoscopy. The working diagnosis was CVID with hepatosplenomegaly and suspected pulmonary involvement. Differential diagnoses included sarcoidosis, other granulomatous diseases, and chronic infections like non-tuberculous mycobacteria and fungal infections (histoplasmosis, coccidioidomycosis, cryptococcosis, aspergillosis). A CT-guided core-needle biopsy of the lung was performed.

**Figure 7:**
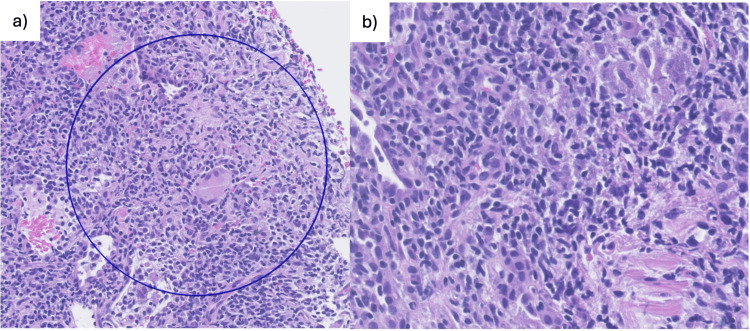
Histopathology of lung biopsy (a) Patchy interstitial lymphocytic infiltrates with nodular arrangements, scattered histiocytes, occasional non-necrotizing granulomas, and rare multinucleated giant cells, consistent with granulomatous–lymphocytic interstitial lung disease (GLILD); (b) Higher-magnification view

Histopathology showed patchy interstitial lymphocytic infiltrates with nodular arrangements, scattered histiocytes, occasional non-necrotizing granulomas, and rare multinucleated giant cells, consistent with GLILD (Figure 7). Immunohistochemistry demonstrated heterogeneous CD3 and CD20 staining. Bronchial histology showed only nonspecific inflammation. Peripheral B-cell immunophenotyping revealed marked reductions in memory B cells (switched and unswitched), absence of plasmablasts, and increased CD21^low^ B cells, alongside mild T-cell subset alterations, confirming CVID with a B+smB-21^low^ profile according to the Euroclass classification. The EUROClass classification is a system used to categorize patients with CVID based on detailed B-cell immunophenotyping. Its purpose is to identify biologically meaningful subgroups that correlate with clinical features and complications.

She was initiated on immunoglobulin replacement therapy, beginning with 20 g IV every four weeks for one month, then 20 g SC every four weeks for two months, and is currently receiving 40 g SC every four weeks. She waits for chest and abdominal CT reevaluation, but no respiratory symptoms or new infectious episodes have been observed to date. Further genetic evaluation and pulmonary function tests are pending. This case illustrates a delayed diagnosis of CVID in a young adult, with pulmonary GLILD as the presenting manifestation, accompanied by splenomegaly, lymphadenopathy, and characteristic immunologic findings, highlighting the subtlety and complexity of CVID presentations beyond childhood.

## Discussion

GLILD is a recognized non-infectious complication of CVID that can significantly impact morbidity and mortality [1,3,6,7]. GLILD develops in approximately 10-20% of patients with CVID and is associated with a significantly worse prognosis compared to those without GLILD [2,6,7]. It represents a spectrum of lymphoproliferative and granulomatous pulmonary involvement, including lymphoid hyperplasia, non-necrotizing granulomas, and variable interstitial inflammation [1,7,8]. Radiologically, GLILD manifests as a combination of pulmonary nodules, ground-glass opacities, interlobular septal thickening, and mediastinal or hilar lymphadenopathy [2,3]. These features often overlap with infectious, neoplastic, or sarcoid-like processes, complicating diagnosis [2,3,9].

Our patient illustrates several important aspects of GLILD in CVID. First, she presented non-infectious pulmonary findings, hepatosplenomegaly, and lymphadenopathy after a resolved infection of influenza A. Her childhood history of recurrent infections was subtle, highlighting that CVID diagnosis may be delayed until adolescence or adulthood [7]. Second, the histopathological findings of patchy interstitial lymphocytic infiltrates with scattered non-necrotizing granulomas, heterogeneous T- and B-cell populations, and the presence of CD21^low^ B cells are consistent with prior reports describing GLILD as a lymphoproliferative manifestation with unique immunologic signatures [1,2,10,11].

Recent studies suggest that granulomas in CVID display histopathological differences compared to other granulomatous diseases, including a higher prevalence of mixed lymphoid and histiocytic patterns, lower necrosis, and a distinctive immune cell composition, which was observed in the histopathology of the lung biopsy of our patient [10]. This supports the notion that GLILD represents a disease-specific manifestation of immune dysregulation rather than a generic granulomatous response.

Management strategies for GLILD remain heterogeneous. Corticosteroids are commonly used for induction, whereas immunomodulators and B-cell-targeted therapies, such as rituximab, are considered in refractory cases or to reduce steroid burden [4,9]. Immunoglobulin replacement therapy is critical for preventing infections, though it seems not to directly modify GLILD pathology [4,7,12]. There is currently no agreement on whether simply monitoring patients after achieving adequate immunoglobulin replacement levels constitutes an appropriate management approach for GLILD [4,12]. Longitudinal follow-up is essential, as pulmonary involvement can progress insidiously even in the absence of overt infections, and non-infectious complications such as splenomegaly or lymphadenopathy may precede significant respiratory symptoms [3,5].

In summary, this case underscores the need for heightened clinical awareness of GLILD as a possible first manifestation of CVID in young adults. Detailed imaging, immunologic profiling, and histopathological confirmation are pivotal for accurate diagnosis. Early recognition enables the timely initiation of immunoglobulin replacement, targeted therapy, and close monitoring, potentially improving long-term outcomes.

This report has several limitations. First, as a single-case description, the findings cannot be generalized to the broader population of patients with CVID or GLILD. Second, genetic testing is still pending, which limits the ability to fully characterize the underlying immunodeficiency and its implications for management [8,13]. Finally, the short duration of follow-up does not allow for a reliable assessment of GLILD progression or stability.

## Conclusions

This case demonstrates that imaging findings disproportionate to the severity of an acute influenza infection can unmask underlying immunodeficiencies, facilitating the diagnosis of CVID and its complication GLILD, in a young adult. Careful review of her childhood history, which included recurrent infections previously overlooked, was crucial in supporting the diagnosis. Clinicians should remain vigilant for primary immunodeficiencies when clinical or imaging features are inconsistent with common infections, enabling timely intervention and improved long-term outcomes.
